# Single-Cell RNA and ATAC Sequencing Reveal Hemodialysis-Related Immune Dysregulation of Circulating Immune Cell Subpopulations

**DOI:** 10.3389/fimmu.2022.878226

**Published:** 2022-05-26

**Authors:** Hongwei Wu, Jingjing Dong, Haiyan Yu, Kang Wang, Weier Dai, Xinzhou Zhang, Nan Hu, Lianghong Yin, Donge Tang, Fanna Liu, Yong Dai

**Affiliations:** ^1^ The Second Clinical Medical College of Jinan University, Shenzhen People’s Hospital, Shenzhen, China; ^2^ Institute of Nephrology and Blood Purification, The First Affiliated Hospital of Jinan University, Jinan University, Guangzhou, China; ^3^ College of Natural Science, University of Texas at Austin, Austin, TX, United States

**Keywords:** maintenance hemodialysis, single-cell RNA sequencing, single-cell assaying transposase accessible chromatin sequencing, T cell receptor, antigen presentation, immune pathways

## Abstract

**Background:**

An increased risk of infection, malignancy, and cardiovascular diseases in maintenance hemodialysis patients is associated with hemodialysis-related immunity disturbances. Although defects in T-lymphocyte-dependent immune responses and preactivation of antigen-presenting cells have been documented in hemodialysis patients, the effects of long-term hemodialysis on the transcriptional program and chromosomal accessibility of circulating immune cell subpopulations remain poorly defined.

**Methods:**

We integrated single-cell RNA sequencing (scRNA-seq) and single-cell assay for transposase-accessible chromatin sequencing (scATAC-seq) to characterize the transcriptome profiles of peripheral mononuclear cells (PBMCs) from healthy controls and maintenance hemodialysis patients. Validation of differentially expressed genes in CD4+ T cells and monocytes were performed by magnetic bead separation and quantitative real-time PCR.

**Results:**

We identified 16 and 15 PBMC subgroups in scRNA-seq and scATAC-seq datasets, respectively. Hemodialysis significantly suppressed the expression levels of T cell receptor (TCR) genes in CD4+ T cell subsets (e.g., TRAV4, CD45, CD3G, CD3D, CD3E) and major histocompatibility complex II (MHC-II) pathway-related genes in monocytes (HLA-DRB1, HLA-DQA2, HLA-DQA1, HLA-DPB1). Downstream pathways of TCR signaling, including PI3K-Akt-mTOR, MAPK, TNF, and NF-κB pathways, were also inhibited in CD4+ T cell subpopulations during the hemodialysis procedure. Hemodialysis altered cellular communication patterns between PBMC subgroups, particularly TGF-TGFBR, HVEM-BTLA, and IL16-CD4 signalings between CD4+ T cells and monocytes. Additionally, we found that hemodialysis inhibited the expression of AP-1 family transcription factors (JUN, JUND, FOS, FOSB) by interfering with the chromatin accessibility profile.

**Conclusions:**

Our study provides a valuable framework for future investigations of hemodialysis-related immune dysregulation and identifies potential therapeutic targets for reconstituting the circulating immune system in maintenance hemodialysis patients.

## Introduction

As an alternative treatment for patients with end-stage renal disease, hemodialysis (HD) patients exhibit a chronic inflammatory state, elevated oxidative stress, and aberrant activation/senescence of blood immune cells, which is associated with chronic stimulation of uremic toxins, malnutrition, and electrolyte disturbances (1, 2). Additionally, dialysis-related factors, such as chronic exposure of blood to synthetic materials of dialysis filters and hemodynamic abnormalities (3), accelerate the imbalance between pro-inflammatory and anti-inflammatory mechanisms, leading to an increased risk of infection, cardiovascular disease, and malignancy in hemodialysis patients (3–5). Mortality from respiratory infections is more than 16 times higher in HD patients than in the general population (6). Immune system maintenance is recognized as one of the therapeutic targets for reducing mortality in maintenance hemodialysis (MHD) patients (3). Although efforts have been made to improve the biocompatibility and solute clearance of dialysis membranes to reduce the immune response caused by recurrent contacts between peripheral blood mononuclear cells (PBMC) and the dialysis membrane (7), long-term hemodialysis remains a highly immune-disruptive and pro-inflammatory process.

Characteristics of immune system dysfunction associated with uremia and “dialysis syndrome” have been reported (3, 8–12). From the innate immune response perspective, hemodialysis activates the complement system by affecting the alternative and lectin pathways (13), leading to an abnormal release of immuno-inflammatory factors. Besides, the chronic hemodialysis procedure causes dysregulation of CD4+/CD8+ lymphocytes (14, 15), a diffuse reduction in B cells (10), and functional inactivation of circulating lymphocytes (12). However, little attention has been paid to the effects of hemodialysis on the transcriptional profile and cellular communication of blood immune cell subpopulations. As a new cutting-edge technique, single-cell RNA sequencing (scRNA) can accurately identify different cell types in tissues through unsupervised clustering and provide a comprehensive gene expression matrix for each cell subset (16). From an epigenetic perspective, a single-cell assay for transposase-accessible chromatin using sequencing (scATAC) can provide robust information on the regulation of gene expression programs by identifying open regions of chromosomes (17). In the present study, we integrated scRNA-seq and scATAC-seq to characterize the immune dysfunction of PBMC in the hemodialysis environment, revealing the mechanisms behind the immune disorders specific to hemodialysis and providing a perspective for improving the prognosis of HD patients.

## Methods

### Study Design

Ten healthy controls (female = 5, mean age: 48.8 ± 11.86 years) and ten HD patients (female = 5, mean age: 49.3 ± 11.62 years, dialysis age: 7.4 years) were recruited to perform scRNA-seq and scATAC-seq experiments. PBMCs from three healthy controls (female = 2, mean age: 43.2 ± 8.31 years) and three HD patients (female = 2, mean age: 51.9 ± 7.21 years, dialysis age: 6.8 years) were collected to perform bulk RNA sequencing. Patients with recent infections, immune diseases, liver disease, and malignancies were excluded. All participants had not been treated with antibiotics, non-steroid anti-inflammatory drugs, and immunosuppressants for at least two months. The study was approved by the Ethics Committee of the Shenzhen People’s Hospital, and the study protocol adhered to the Declaration of Helsinki. All participating individuals have signed informed consent. Clinical characteristics are summarized in **Supplementary Table 1**.

### Nuclei Suspension Preparation and Single-Cell Library Construction

Blood samples (3 ml per subject) were diluted with equal volumes of PBS. PBMCs were separated using an equal proportion of Ficoll-Paque Plus. We used 1 ml of PBS + 0.04% BSA to resuspend cell pellets and then passed cell suspensions through a 40-µm Flowmi Cell Strainer. The hemocytometer was applied to determine the cell concentration. We added PBMCs to a 2 ml microcentrifuge tube and used 100 µl chilled lysis buffer (10 mM Tris-HCl, 3 mM MgCl2, 10 mM NaCl, 0.1% Tween-20, 0.1% Nonidet P40 Substitute, 1% BSA) to obtain a final homogeneous nuclei suspension. For scRNA-seq and scATAC-seq data, the ten cellular suspensions from HD patients and healthy controls were separately pooled with an equal volume and then diluted to a 1 x 10^6^ cell/mL concentration. Notably, there was at least a slight donor bias in the number of cells from each sample in the mixture. The mixture suspension was then loaded onto the 10X Genomics platform to create a barcoded cDNA library for individual cells (18). Data quality control was performed using the Bioanalyzer (Agilent). Following quantification with the KAPA Library Quantification Kit (Roche), individual libraries were pooled for sequencing on the HiSeq 2500 (Illumina).

### ScRNA-Seq Quantification and Data Analysis

Raw FASTQ files were matched to the hg38 reference genome, and gene quantification was performed by CellRanger (version 3.1.0) to generate filtered gene-barcode matrices. We used the count matrix to establish a Seurat object. The standard processing workflow (e.g., quality control, data normalization, and scaling) for the scRNA-seq data was performed by the Seurat R package (V3.1.2) (19). Poor-quality cells with unique feature counts < 200 or > 2500, UMI < 30000, and > 5% mitochondrial counts were filtered, leaving 12367 HC-PBMC and 8780 HD-PBMC cells for further analytical processing. The data removing unqualified cells was further normalized by the global-scaling normalization method “LogNormalize”. We used the FindVariableFeatures function to identify highly variable genes. By default, 2000 features were returned per dataset. After a linear transformation performed by ScaleData, we used principal component analysis (PCA) to perform the linear dimensional reduction. JackStrawPlot was applied to evaluate the “dimensionality” of the dataset. The top 15 principal components were utilized in projection (UMAP) visualization and clustering in our data. Cell clustering was done using the FindClusters function with a resolution of 1.2, which resulted in 16 distinct cell clusters. The FindMarkers function with the default parameters (Benjamini-Hochberg adjusted *P*-value < 0.05 in Wilcoxon rank-sum test, |Log2 FC| > 0.25) was used to identify differentially expressed genes between cell clusters.

### ScATAC-Seq Quantification and Data Processing

The scATAC-seq library preparation was performed as recommended by the Chromium Single Cell ATAC Reagent Kits User Guide (10x Genomics). The “CellRanger-ATAC mkfastq” pipeline was applied to convert the raw sequencing data to the FastQ file. scATAC-seq reads were matched to the GRCh38 reference genome and quantified using the “CellRanger-count” pipeline. We used the ArchR package (20) to process the fragment files and create a genome-wide TileMatrix using 500-bp bins. Poor-quality cells with transcription start site (TSS) enrichment score < 4 and unique nuclear fragments < 1000 were filtered, leaving 7556 HC-PBMC and 6591 HD-PBMC cells for further data processing. The average TSS scores for the HC and HD samples were 14.3 and 10.8, respectively, indicating the high quality of these scATAC-seq datasets. Dimensionality reduction was performed by iterative latent semantic indexing (LSI), followed by batch effect correction with Harmony. Clustering was performed using Seurat’s FindClusters function with a resolution of 1.2, generating 15 scATAC-seq cell subpopulations. We applied two different approaches to annotate cell clusters. First, we calculated gene scores following the default in ArchR and used the getMarkerFeatures function to identify marker features for each cell cluster, followed by marker gene visualization on our Harmony UMAP embedding. Besides, ArchR enables integration with scRNA-seq to define cluster identity. Unconstrained and constrained integration methods were used to align scATAC-seq cells with scRNA-seq cells by comparing the gene expression matrix from the scRNA-seq data with the gene score matrix from the scATAC-seq data.

Bedtools merge (version 2.26.0) and MACS2 were applied to identify a union set of variable-width accessible regions and peaks, respectively. Marker peaks for each PBMC subset were identified by calling the addMarkerFeatures function. Differential peaks between groups were defined as peaks with an adjusted *P* value < 0.05 and |Log2FC| > 0.25. After establishing a robust peak set, we performed motif enrichment in differential peaks and marker peaks to discover the enrichment of key TFs in cell type-specific accessible chromatin regions. We next used the TF footprinting method to predict the precise binding location of *FOS*, *FOSB*, *JUN*, and *JUND* at a particular locus in CD4+ TEM and naive CD4+ T cells. After obtaining the location of the relevant TF motifs with the getPositions function, we can plot these TF footprints using the plotFootprints function. Normalization of footprints for Tn5 Bias was conducted by setting normMethod = “Divide”.

### Cell-Cell Communication Analysis Between PBMC Subpopulations

For scRNA-seq data, we used CellChat (21) to identify the ligand-receptor interaction and cell-cell communication between PBMC subpopulations. The process and description of CellChat analysis are detailed at https://github.com/sqjin/CellChat. Significant ligand-receptor pairs between cell types were defined as those with a *P*-value < 0.05.

### Pathways and Gene Ontology Terms Associated With Hemodialysis

Gene set enrichment analysis (GSEA) was performed using the Clusterprofiler package (22). The predefined gene set MSigdb is available in https://www.gsea-msigdb.org/gsea/msigdb/index.jsp. Differentially expressed genes (adjusted *P* value < 0.05 and |Log2FC| > 0.25) from each PBMC subpopulation were put into the DAVID online software for kyoto encyclopedia of genes and genomes (KEGG) and gene ontology (GO) analysis (https://david.ncifcrf.gov/). Pathways and biological processes with *P* values < 0.05 were considered significantly enriched.

### Validation of Critical Genes Associated With the TCR Signaling Pathway Using RT-qPCR

Five HD patients (mean age: 41.3 ± 5.3 years, dialysis age: 5-8 years) and three age-matched healthy subjects were recruited. PBMCs were isolated using ficoll-hypaque density gradient centrifugation. CD4+ T cells and monocytes were purified by positive selection with magnetic-activated cell separation CD4+ and CD14+ immunomagnetic beads (Miltenyi), respectively. RNA was extracted from CD4+ T cells and monocytes using RNAiso Plus, isopropanol, and chloroform, followed by reverse transcription using PrimeScript RT Master Mix (Takara, RR036A). The cDNA was then utilized in RT-qPCR. The primer sequences for the genes are as follows: *AKT1* forward: CCGCTACTACGCCATGAAGATC, reverse: CGGTTCTCGGTGAGTGTGTG; *JAK1* forward: CTGTCCTGGCCATCTCACAC, reverse: GGTGAGAAGGTTCCTCTGTCTG; *PIK3CA* forward: CACCTGAATAGGCAAGTCGAG, reverse: CCTGTAGAGCATCCATGAAATCTG; *FOS* forward: GGAGGGAGCTGACTGATACAC, reverse: AGCTGCCAGGATGAACTCTAG; *JUN* forward: GAGAGCGGACCTTATGGCTAC, reverse: GTGAGGAGGTCCGAGTTCTTG; *mTOR* forward: GAGAGGCCATCCGTGTGTTAG, reverse: ACTTGGATTCTGACAGGCTGAC; IFI30 forward: CCTGCGTGTTGGATGAACTTG, reverse: CAGGCATAGTGGCAGACTTCTC; HLA-DQA1 forward: GCTCTGACCACCGTGATGAG, reverse: GCAGTCTCCTTCCTCTCCAG; HLA-DQA2 forward: CTCTACCGCTGCCACCAATG, reverse: GCTCAGCCAGGTGATGTTGAC; HLA-DRB1 forward: CCTGACGCTGAGTACTGGAAC, reverse: CCGTAGTTGTGTCTGCAGTxAGG.

### Statistical Information

GraphPad Prism 7 was used for statistical analysis. For RT-qPCR data, Student’s t-test was used to compare genes between groups, and *P* values < 0.05 were considered significantly changed. The differentially expressed genes were analyzed using the Wilcoxon rank-sum test. The Benjamini-Hochberg test was used to correct the statistical *P*-value.

## Results

### Characteristics of Immunological Genetic Changes in PBMC in Patients Receiving MHD

**Figure 1:**
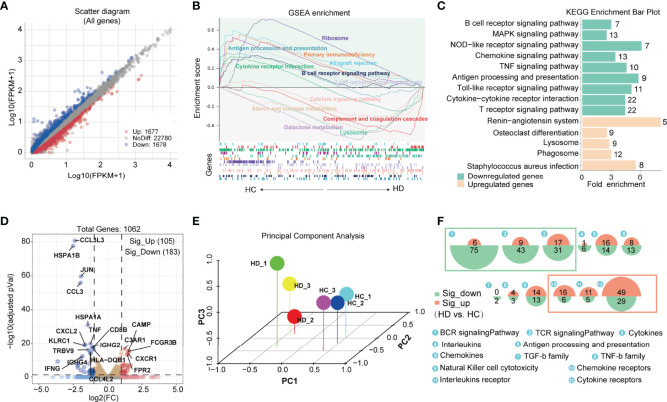
Features of immunological genetic changes in PBMC of HD patients based on bulk RNA-seq data. **(A)** Identification of differentially expressed genes (DEGs) between HC and HD groups. Adjusted *P* value < 0.05 and |Log2FC| > 0.25 were used as screening criterion. Blue and red dots represent down-regulated and up-regulated genes in the HD group, respectively. **(B)** Gene set enrichment analysis of DEGs. Up: curves of the change (increase/decrease) in the enrichment score values of the predefined gene sets in different functional units across the cumulative process. Each line indicates one functional unit marked with a specific color; Down: location of the gene members of the predefined gene set in the functional units, with arrows showing the direction of enrichment in the HD and HC groups. Functional units associated with immune function (*P* < 0.05) are presented in the picture. **(C)** KEGG analysis of down-regulated and up-regulated genes. The green and orange bars show the pathways enriched by the down-regulated and up-regulated genes, respectively. The Arabic numbers on the right indicate the number of differential genes in the corresponding pathway. **(D)** Volcano plot showing immune-related genes differentially expressed between groups. Red dots and blue dots represent up-regulated and down-regulated immune genes in the HD group, respectively. **(E)** Principal component analysis based on the immune gene matrix. One dot represents one sample. **(F)** Proportional area chart showing the number of differential genes in the corresponding immuno-inflammatory pathway. The areas of the green and orange circles show the number of down-regulated and up-regulated genes, respectively. PBMC, peripheral blood mononuclear cell; DEGs, differentially expressed genes; KEGG, Kyoto encyclopedia of genes and genomes; HD, hemodialysis; HC, healthy control.

We performed bulk RNA sequencing to establish a comprehensive transcriptome profile of PBMCs under hemodialysis. A total of 26135 genes were discovered, with 1677 genes up-regulated and 1678 genes down-regulated in hemodialysis patients (**Figure 1A**). To gain greater understanding of the biological functions performed by all these identified genes, we utilized GSEA analysis to calculate the enrichment scores of potential pathways. The results revealed that complement and coagulation cascades, galactose metabolism, starch and sucrose metabolism, and the calcium signaling pathway were significantly enriched in HD patients, while B and T cell receptor signaling pathways and the antigen processing process were significantly attenuated (**Figure 1B**). In keeping with GSEA, KEGG analysis also showed disturbances in B and T cell receptor signaling pathways and antigen processing and presentation in HD patients (**Figure 1C**). We then analyzed the effect of hemodialysis on immune-related genes that were provided by the IMMPORT database (https://www.immport.org/shared/genelists). A total of 1062 immune-related genes were identified in our RNA-seq data, with 183 genes significantly down-regulated and 105 genes up-regulated in HD patients (**Figure 1D**). PCA clustering performed with this immune gene matrix showed a clear separation of samples between groups (**Figure 1E**), suggesting that the transcriptional profile of hemodialysis-associated PBMC was distinct from that of healthy subjects. Importantly, genes curated with functions and gene ontology terms indicated that hemodialysis primarily interfered with the expression of genes associated with immune cell receptor signalings, for example, genes for B cell and T cell receptor signalings were significantly reduced, while genes for chemokine, interleukins, and cytokine receptor signalings were predominantly increased (**Figure 1F** and **Supplementary Table 2**).

### The Cell Type Annotation of scRNA-Seq and scATAC-Seq Clusters

**Figure 2:**
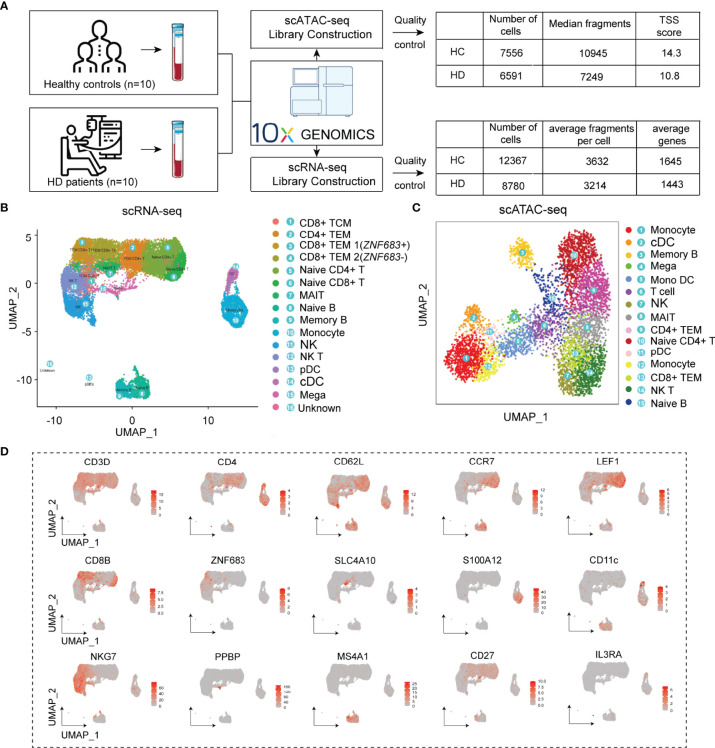
Cell type identification of scRNA-seq and scATAC-seq clusters. **(A)** Flowchart of the study and results of data quality control. **(B)** UMAP plots visualizing clusters of PBMCs derived from scRNA-seq data. **(C)** UMAP plot visualizing clusters of PBMCs derived from scATAC-seq data. **(D)** UMAP plots showing representative marker gene expression levels in the PBMC subpopulations. PBMC, Peripheral blood mononuclear cell; UMAP, uniform manifold approximation and projection for dimension reduction.

We generated scRNA-seq and scATAC-seq profiles for PBMC samples from HC and HD patients to obtain specific transcriptional profiles for each immune cell subpopulation in the hemodialysis environment. After rigorous quality control (**Figure 2A** and **Supplementary Figure 1**), 21147 cells were clustered into 16 PBMC subgroups in the scRNA-seq data (**Figure 2B**). Based on known marker genes (**Supplementary Table 3**) (23–25), these clusters were defined as CD8+ central memory T cells (CD8+ TCM, Cluster 1: *CD8A*+, *CD8B*+, *CD3D*+, *CCR7*+, *CD62L*+); CD4+ effector memory T cells (CD4+ TEM, cluster 2: *CD4*+, *CD3D*+, *CD62L*-, *CCR7*-); *ZNF683*+ CD8+ TEM cells (CD8+ TEM1, Cluster 3: *ZNF683*+, *CD8A*+, *CD8B*+, *CD3D*+, *CD62L-*, *CCR7*-); ZNF683- CD8+ TEM cells (CD8+ TEM2, Cluster 4: *ZNF683*-, *CD8A*+, *CD8B*+, *CD3D*+, *CD62L-*, CCR7-); Naive CD4+ T cells (Cluster 5: *CD4*+, *CD3D*+, *CCR7*+, *LEF1*+); Naive CD8+ T cells (Cluster 6: CD8A+, *CD8B* ^+^, *CD3D*+, *LEF1*+); MAIT cells (Cluster 7: *CD8A*+, *CD8B*+, *CD3D*+, *SLC4A10*+); Naive B cells (Cluster 8: *MS4A1*+, *CD19*+, *CD27*-, *IGHD*+); Memory B cells (Cluster 9: *MS4A1*+, *CD19*+, *CD27*+, *IGHG1*+); Monocytes (Cluster 10: *S100A12*+, *CD14*+); Natural killer cells (NK, cluster 11: *NKG7*+, *ZNF683*+, *CD3D*-); NKT cells (Cluster 12: *NKG7*+, *ZNF683*+, *CD3D*+); Plasmacytoid dendritic cells (pDCs, cluster 13: *IL3RA*+, *NRP1*+, *LILRA4*+); Conventional dendritic cells (cDC, cluster 14: *CD11c*+, *S100A12*-); Megakaryocyte (Mega, cluster 15: PPBP+, PF4+). In addition, we annotated 15 cell clusters generated from scATAC-seq data by integrating the scRNA-seq and scATAC-seq matrix (See Method section). We identified these corresponding scATAC-seq clusters as CD4+ TEM, naive CD4+ T cells, CD8+ TEM, cDC, monocytes, B cells, MAIT, and NK cells, closely resembling the annotation result obtained from scRNA-seq analysis (**Figure 2C**). Cell-type-specific marker genes and the proportions of each cell subset are shown in **Figure 2D** and **Supplementary Figure 1**.

### The Hemodialysis-Related Biological Process of PBMC Subpopulations

**Figure 3:**
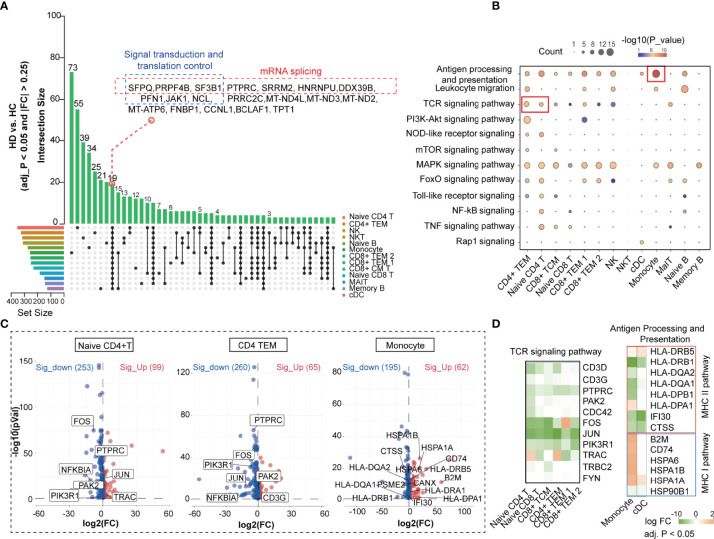
Hemodialysis-related changes in the biological process of PBMC subpopulations based on the scRNA-seq data. **(A)** UpSet plot showing the integrated comparative analysis of DEGs in PBMC subpopulations between HD and HC groups. The horizontal bars on the left show the number of the DEGs for each PBMC subpopulation. Each color represents one PBMC cluster. Individual dots indicate the DEG set specific to a cell subset. The lines between dots indicate the shared DEG set between PBMC subpopulations. The vertical bars indicate the number of DEGs in the corresponding DEG set. **(B)** KEGG analysis of DEGs in PBMC subpopulations. The dot size denotes the number of DEGs in the corresponding pathway. Color bars show *P* values. KEGG analysis is performed by DAVID. **(C)** Volcano plot showing DEGs in CD4+ TEM, naive CD4+ T cells, and monocytes. **(D)** Heatmap showing expression levels of the key DEGs in the corresponding PBMC subpopulations. The color bar shows FC values. PBMC, peripheral blood mononuclear cell; DEGs, differentially expressed genes; KEGG, Kyoto encyclopedia of genes and genomes; DAVID, database for annotation, visualization and integrated discovery; FC, fold change.

We obtained differentially expressed genes (DEGs) from each PBMC subset in the HC and HD groups to establish a cell-subtype-specific gene signature in HD patients (**Supplementary Table 4**). Comparison of the number of DEGs showed that hemodialysis exhibited the greatest effect on the transcriptome profile of naive CD4+ T cells and CD4+ TEM, followed by NK and NKT cells (**Figure 3A**). We found 19 differential genes, including hub genes involved in mRNA splicing (*SFPQ*, *PRPF4B*, *SF3B1*, *PTPRC*, *SRRM2*, *HNRNPU*, *DDX39B*) and translation regulation processes (*PFN1*, *JAK1*, *NCL*) (26, 27), that were commonly changed in all PBMC subpopulations, implying a remarkable effect of hemodialysis on gene expression programming across all immune cell subgroups. To better understand the impact of hemodialysis on the biological processes of PBMC subgroups, we assessed the enrichment degree of immune- and inflammation-related pathways for each PBMC subset based on the DEG matrix. Pathway enrichment analysis showed that the TCR signaling pathway was aberrant in CD4+ TEM and naive CD4+ T cells in HD patients, while monocytes were characterized by dysregulated antigen presentation processes (**Figure 3B**). Notably, the expression levels of TCR signaling-related genes (*FOS*, *JUN*, *PTPRC*, *PAK*2, *NFKBIA*, *PIK3R1*) were suppressed in dialysis-derived naive CD4+ T cells and CD4+ TEM (**Figure 3C**). MHC-II pathway-related genes (*HLA-DRB1*, *HLA-DQA2*, *HLA-DQA1*, *HLA-DPB1*, *IFI30*, *CTSS*) showed a decreasing trend in antigen-presenting cells (APCs), whereas MHC-I pathway-related genes (*B2M*, *CD74*, *HSPA6*, *HSPA1B*, *HSPA1A*) displayed the opposite tendency (**Figures 3C, D**). These findings suggested that hemodialysis might inhibit the TCR-pMHCII signaling between APCs, mainly monocytes, and CD4+ T cells (12). Considering the critical role of classical TCR-pMHCII interactions in initiating antigen-specific immune responses in CD4+ T cells (28), we speculated that hemodialysis might affect cell-cell communication signaling among PBMC subpopulations. Therefore, we performed CellChat analysis to characterize cellular communication between blood immune cells in the hemodialysis environment.

### Features of Cell-Cell Communication Patterns in HD Patients

**Figure 4:**
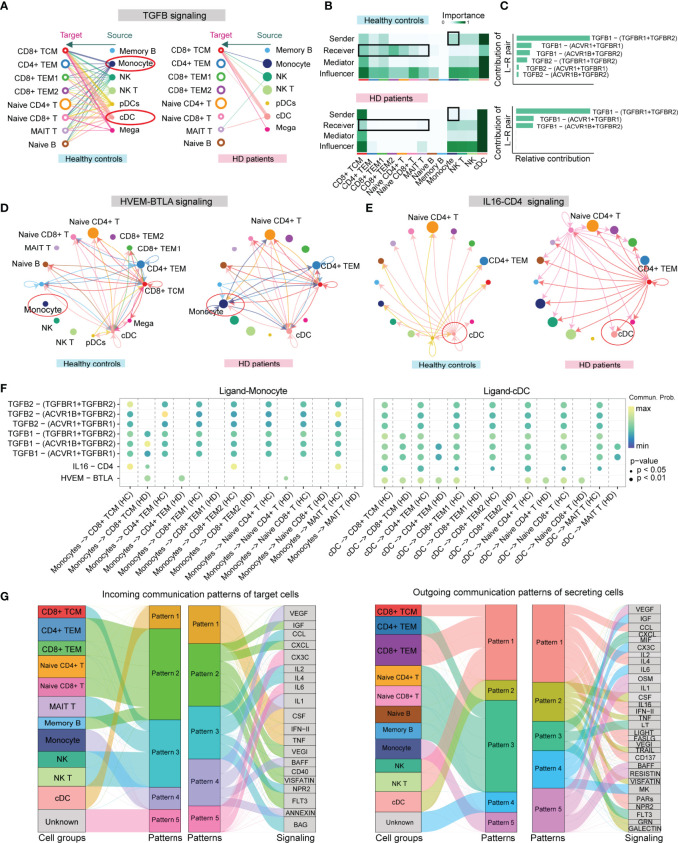
CellChat analysis of the communication between PBMC subpopulations in HD patients. **(A)** Hierarchical plot showing the inferred cell-cell communication network for TGFB signaling. Open and solid circles denote target (receptor) and source (ligand), respectively. Edge width represents the communication probability. **(B)** Heatmap showing the relative importance of each PBMC subpopulation based on the computed four network centrality measures (sender, receiver, mediator, and influencer) of the TGFB signaling network. The color bar represents important values. **(C)** The relative contribution of each ligand-receptor pair to the overall communication network of the TGFB signaling pathway. **(D)** The inferred HVEM-BTLA signaling network in HD and HC groups. Arrows show signal transmission direction from the source to the target. **(E)** The inferred IL16-CD4 signaling network in HD and HC groups. **(F)** Comparison of the significant ligand-receptor pairs of TGFB, BTLA, and CD4 signaling pathways between the HD and HC groups, which contributes to the signaling from APCs (DC and monocyte) to T cell subpopulations. The color bar reflects communication probabilities. Dot size represents *P* values. A *P-*value is computed from a one-sided permutation test. **(G)** Alluvial plots show the incoming signaling patterns of target cells (left) and the outgoing signaling patterns of secreting cells (right) in HD patients. The thickness of the flow represents the contribution of the cell group or signaling pathway to each corresponding pattern. PBMC, peripheral blood mononuclear cell; APCs, antigen-presenting cells; cDC, conventional dendritic cell.

Compared to other methods (e.g., CellPhone and iTalk) for inferring cell-cell communication using only one ligand/receptor pair, CellChat allows researchers to specifically explore the role of multisubunit complexes of receptors (21). In our scRNA-seq data, CellChat detected 74 ligand-receptor pairs among 15 PBMC subpopulations, which were distributed to 45 signaling pathways, including *CD40*, *CXCL*, *TGFB*, *IL-1*, *TNF*, *BAFF*, and B- and T-lymphocyte attenuator (*BTLA*) pathways (**Supplementary Figure 2A**). By counting the number of ligand-receptor pairs, we found that cellular communication between PBMC subgroups was attenuated in HD patients (**Supplementary Figure 2B**). TGFB signaling between APCs and T cell subsets was remarkably weakened in HD patients (**Figure 4A**). Network centrality analysis of the TGFB signaling revealed that CD4+ TEM, CD8+ TEM, naive CD4+ T, and naive CD8+ T cells were the predominant signal recipients of APCs-derived TGFB ligands under normal homeostasis, but this cellular interaction appeared to be disrupted during hemodialysis (**Figures 4A, B**). Notably, analysis of the relative contribution of each TGFB ligand-receptor pair showed that hemodialysis-induced TGFB signaling inhibition mainly resulted from the attenuation of *TGFB2* and its multimeric *TGFBR1*, *TGFBR2*, *ACVR1*, and *ACVRB1* receptors (**Figure 4C**). In addition to affecting the TFGB signaling, hemodialysis stimulated the HVEM-BTLA signaling between monocytes and CD4+ T cell subpopulations (CD4+ TEM, naive CD4+ T cell, and CD8+ TCM) (**Figure 4D**), while inhibited the IL16-CD4 signaling between DCs and all T cell subsets (**Figure 4E**). In addition, we observed that IL16-CD4 outgoing signaling appeared to be selectively dysregulated in CD8+ TCM, implying that hemodialysis significantly altered the IL16 secretion pattern in CD8+ TCM. The value of ligand-receptor pairs of TGFB, BTLA, and CD4 signalings sending from APCs to each T cell subpopulation is shown in **Figure 4F**. Of note, TGFB, BTLA, and CD4 signalings have been identified as essential pathways affecting T cell activation, proliferation, and immune function (29–31), suggesting that hemodialysis might suppress T cell activation by APCs through specific costimulatory molecules (31). Finally, we characterized the global communication patterns of PBMC subpopulations in HD patients based on the pattern recognition method (see Method section) (**Figure 4G** and **Supplementary Figure 2C**). Five key patterns for incoming signaling (treating cells as receivers) and five patterns for outgoing signaling (treating cells as senders) were identified. As expected, hemodialysis altered the cellular signal patterns of PBMC subgroups. For example, the incoming signaling of TEM CD4+ and naive CD4+ T cells in the normal milieu was characterized by pattern 4, representing such pathways as *IL6*, *LIFR*, *OSM*, and *FLT3* signalings (**Supplementary Figure 2C**), but this pattern was changed to pattern 2, dominated by *IGF*, *CXCL*, *VEGI*, and *VISFATIN* signalings after receiving hemodialysis (**Figure 4G**).

### Transcriptional Regulation of Naive CD4+ T Cells in the Hemodialysis Environment

**Figure 5:**
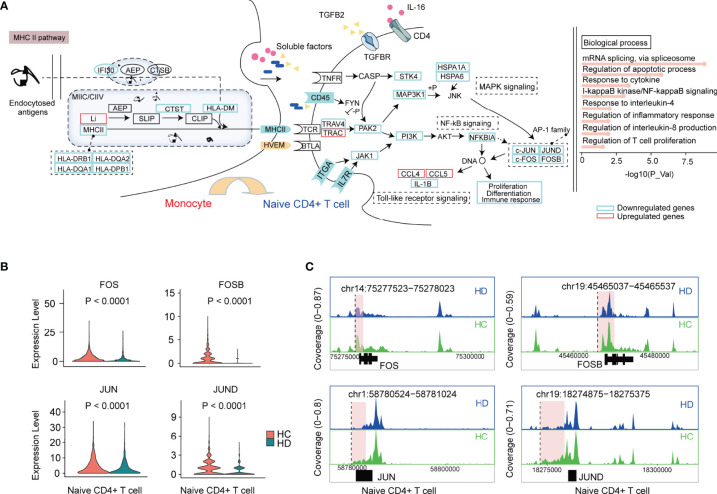
The characteristic of the transcriptional regulation of naive CD4+ T cells in HD patients. **(A)** The schematic illustration of cellular communication between monocytes and naive CD4+ T cells in HD patients. In brief, dysfunctional TCR-pMHCII, TGFB, CD40, and BTLA signalings lead to changes in TCR downstream pathways and biological processes. Genes significantly downregulated and upregulated in naive CD4+ T cells from HD patients are marked with blue and red boxes, respectively. **(B)** Violin plots showing the differential expression of AP-1 family TFs (*FOS*, *FOSB*, *JUN*, *JUND*) in naive CD4+ T cells between HD and HC groups. **(C)** Mean scATAC-seq coverage at *FOS*, *FOSB*, *JUN*, *JUND* locus in naive CD4+ T cells. Differential peaks are marked with red boxes. TCR, T cell receptor; MHCII, major histocompatibility complex II. AP-1, activator protein 1; TFs, transcription factors.

As shown in **Figure 3B**, apart from TCR signaling, downstream pathways of TCR such as MAPK, TNF, and NF-κB signaling pathways were disrupted in naive CD4+ T cells in the hemodialysis environment. Strikingly, most genes involved in these pathways (e.g., *JAK1*, *PAK2*, *PIK3R1*, *NFKBIA*, *JUND*, *FOS*, *FOSB*) showed a decreasing trend (**Figure 5A** and **Supplementary Table 4**). Based on pathway enrichment and cell-cell communication analyses, we suggested that the attenuation of these TCR downstream pathways could be partly attributed to TCR-pMHCII dysfunction, since hemodialysis remarkably suppressed expression levels of membrane receptors (*CD45*, *ITGA*, *IL7R*, *TRAC*, *TRAV*) on naive CD4+ T cells, and MHC-II pathway-related genes (*HLA-DRB1*, *HLA-DQA2*, *HLA-DQA1*, *HLA-DPB1*) on monocytes (**Figure 5A**). A decrease in costimulatory signals involving TGFB2-TGFBR and IL16-CD4 and an increase in HVEM-BTLA signaling might negatively impact naive T cell activation. Gene ontology analysis conducted by DAVID revealed abnormalities in the biological processes of mRNA splicing, apoptosis, response to interleukins and cytokines, and regulation of T cell proliferation in naive CD4+ T cells (**Figure 5A**). We cannot rule out the possibility that the changes in these biological behaviors are closely related to the dysfunction of TCR-pMHCII and its downstream signaling pathway.

Among all these altered genes, the decreased levels of AP-1 family transcription factors (*JUN*, *JUND*, *FOS*, *FOSB*) caught our attention because they were reported to directly regulate T cell proliferation, differentiation, and immune response by affecting DNA expression (**Figures 5A, B**) (32, 33). However, scRNA-seq provides limited information on transcription factors (TFs), making it challenging to unravel how genes are regulated. Therefore, in parallel, we used scATAC-seq to generate an open chromatin map to pinpoint the availability of TF binding sites and to identify the gene regulatory logic. 14535 differentially accessible open chromatin peaks (DAPs) were obtained across PBMC subpopulations (adjusted *P* < 0.05 and |Log2FC| > 0.58), of which 532 DAPs were identified in naive CD4+ T cells (**Supplementary Table 5** and **Supplementary Figures 3A, B**). TF motif accessibility analysis (see Method section) showed that *FOS*, *JUN*, and *JUND* motifs were enriched in CD4+ TEM and naive CD4+ T cells, highlighting an essential regulatory role of AP-1 family TFs in CD4+ T cells (**Supplementary Figures 3C, D**). However, comparative analysis of TF footprints showed that the enrichment of these TFs at the transcription start site did not appear to differ between the HC and HD groups (**Supplementary Figure 3E**). Interestingly, we observed a series of DAPs in the vicinity or/and distal elements of *FOS* (chr14:75277523-75278023), *FOSB* (chr19:45465037-45465537), *JUN* (chr1:58780524-58781024), and *JUND* (chr19:18274875-18275375), suggesting that hemodialysis might affect gene expression by interfering with gene cis-regulatory elements (**Figure 5C**).

### Transcriptional Regulation of CD4+ TEM in the Hemodialysis Environment

**Figure 6:**
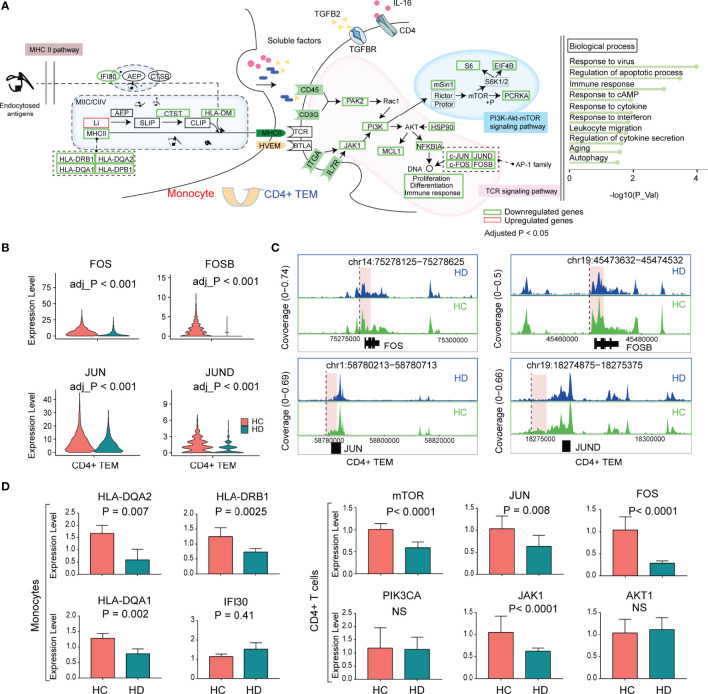
The characteristic of the transcriptional regulation of CD4+ TEM in HD patients. **(A)** The schematic illustration of cellular communication between monocytes and CD4+ TEM cells in HD patients. **(B)** Violin plots showing the differential expression of AP-1 family TFs (*FOS*, *FOSB*, *JUN*, *JUND*) in CD4+ TEM between HD and HC groups. **(C)** Mean scATAC-seq coverage at *FOS*, *FOSB*, *JUN*, *JUND* locus in CD4+ TEM. Differential peaks are marked with red boxes. **(D)** Histograms showing the expression levels of genes validated by RT-qPCR. AP-1, activator protein 1; TFs, transcription factors; RT-qPCR, quantitative real-time polymerase chain reaction.

Resembling naive CD4+ T cells, CD4+ TEM was also characterized by TCR-pMHCII dysfunction in HD patients. We found that the hemodialysis process primarily inhibited the PI3K-Akt-mTOR pathway of TCR downstream signaling, since *ITGA*, *IL7R*, *JAK1*, *PI3K*, *MAPKAP1*, *RPS6*, *EIF4B*, and *PCRKA* showed significantly decreased levels in HD patients. The altered biological processes of CD4+ TEM were dominated by aging, autophagy, and response to cytokines (**Figure 6A**). AP-1 family TFs (*JUN*, *JUND*, *FOS*, *FOSB*) were significantly reduced in CD4+ TEM in HD patients (**Figure 6B**). Notably, we also observed decreased levels of *JUN* and *FOS* in other T cell subpopulations such as CD8+ TEM and naive CD8+ T cells (**Figure 3D**). To better understand the epigenetic alteration in genes, we obtained 956 DAPs in CD4+ TEM (**Supplementary Figure 4A** and **Supplementary Table 5**). Interestingly, most DAPs were enriched in the vicinity or/and distal elements of *FOS* (chr14:75278125-75278625), *FOSB* (chr19:45473632-45474532), *JUN* (chr1:58780213-58780713), and *JUND* (chr19:18274875-18275375) (**Figure 6C** and **Supplementary Figures 4B, C**).

We used RT-qPCR to verify the expression levels of downstream genes (*AKT1*, *JAK1*, *PIK3CA*, *FOS*, *JUN*, and *mTOR*) of the TCR pathway in CD4+ T cells and MHCII-related genes (HLA-DQA1, HLA-DQA2, IFI30, and HLA-DRB1) in monocytes. Compared to the HC group, HD patients showed reduced *JAK1*, *FOS*, *JUN*, *and mTOR* levels in CD4+ T cells, and decreased levels of HLA-DQA1, HLA-DQA2, and HLA-DRB1 in monocytes, which was consistent with the scRNA-seq data (**Figure 6D**).

## Discussion

Chronic suppression or activation of the immune system by hemodialysis is strongly associated with high rates of infection, malignancy, and mortality in HD patients. Although undesirable effects of maintenance hemodialysis on PBMCs have attracted attention, the underlying mechanisms are poorly understood or remain controversial to date. Our study aims to characterize changes in transcriptional profile and hemodialysis-related biological processes of PBMC subpopulations during the long-term hemodialysis procedure by integrating scRNA-seq and scATAC-seq data. We highlight that 1) maintenance hemodialysis (> 5 years) reprogram PBMCs landscape and biological processes; 2) hemodialysis-related monocytes have a weak antigen-presenting ability; 3) Disturbances in TCR-pMHCII and cell-cell communication may lead to dysregulation of immune pathways in CD4+ TEM and naive CD4+ T cells; 4) Hemodialysis inhibited the expression levels of AP-1 family TFs (JUN, JUND, FOS, FOSB) by interfering with chromosomal accessibility profiles.

Although hemodialysis is the primary alternative therapy for patients with end-stage renal disease (ESRD), the proliferation response of peripheral blood lymphocytes is diminished in HD patients, possibly due to chronic pro-inflammatory and pro-oxidant procedures, access-related infections, platelet activation, and persistent hemodynamic abnormalities (8–12). The long-term effects of chronic hemodialysis on T cells manifest principally as the inverted CD4+/CD8+ ratio, decreased production of naive T cells accompanied by attenuated proliferation capacity, and premature aging of CD4+ and CD8+ T cells, resulting in deficiencies in the immune response to vaccinations and increased incidence of bloodstream infections (9, 13). Nongnuch and coworkers demonstrated that Online hemodiafiltration (HDF) increased the percentage of CD38+ Naive CD4+ T cells and CD45RA-CCR7+CD8+ TCM cells compared to those patients treated with HD (9). In addition, hemodialysis induced the shift from a non-aging (CD4+CD28+ T cells) to an aging phenotype (CD4+CD28- T cells) compared to peritoneal dialysis, suggesting that dialysis modality influenced T cell function (34, 35). In addition to reduced numbers of T cell subsets, our scRNA-seq data showed decreased expression levels of T-cell identity markers (CD3E, CD3D) and surface receptors of TCR signaling (e.g., TRAV4, CD45, CD3G, ITGA, IL7R) in CD4+ TEM and naive CD4+ T cells. Previous studies have shown that TCR density on CD4 T cell surface in healthy individuals decreased by 40% after the addition of serum from HD patients. Of clinical relevance is the observation that TCR density on T cell surface correlates with the immune response to hepatitis B virus vaccination in HD patients (36). Thus, diminished expression of these cell membrane receptors may be the key feature of the hemodialysis-associated decline of CD4+ T cell function, which can serve as clinical indicators for monitoring the acquired immunity system in HD patients. Notably, it has been reported that uremia-derived CD4+T cells had a reduced number of TCR/CD3 antigen receptor complexes and a skewed T cell receptor Vβ repertoire (37). Therefore, further investigation is needed to clarify whether the reduction in these receptor levels is driven by uremic toxins or the dialysis procedure.

One important finding of our study, which has not been reported before, is the disorder of immuno-inflammatory pathways of TCR downstream signaling in T cells in HD patients. We found that hemodialysis inhibited downstream pathways of TCR signaling, such as the PI3K-Akt-mTOR pathway in CD4+ TEM cells and Toll-like receptor (TLR), TNF, and NF-κB pathways in naive CD4+ T cells, which may be mainly attributable to the decreased levels of TCR receptors. TLR and their signaling mediators like NF-κB play an essential role in chronic inflammation of MHD patients (38). TLR-4, which can recognize uremic toxins and infections, shows reduced levels in HD patients due to the influx of impurities from the dialysate compartment and cytoskeletal stresses from the roller pump (39). PI3K-Akt-mTOR and TNF pathways are strongly associated with the proliferation of naive and memory T cells (40–42). PI3K/mTOR inhibitor BEZ235 blocks T cell activation by inducing cell cycle arrest in G0/G1 phase (40). However, strong evidence supporting the inhibitory effect of hemodialysis on the mTOR pathway is still lacking, especially data from cellular experiments. Notably, upregulation of serum concentrations of TNFα and NF-κB in HD patients has been observed (43), while our findings show diminished TNF/NK-κB pathway activity in T cells. These data suggest an impaired reaction of hemodialysis-derived T cells to TNFα stimulation. Establishing the interaction of these pathways and identifying common triggers may help provide an efficient approach for treating immune disorders in HD patients. The downregulated expression of shared AP-1 family TFs (*JUN*, *JUND*, *FOS*, *FOSB*) located downstream of these immuno-inflammatory pathways drew our attention, as the AP-1 family is believed to be a critical factor in regulating cell proliferation, survival, differentiation, and T-cell activation (44). Interestingly, We found reduced chromosomal accessibility of the *JUN*, *JUND*, *FOS*, and *FOSB* gene regions in hemodialysis-derived CD4+ T cells, suggesting that hemodialysis affects gene expression by interfering with epigenetic features. AP-1 may be a potential therapeutic target for rescuing T cell function in HD patients.

The defect in T lymphocyte function can be attributed to impaired APC function, as reduced T cell activation occurs after antigen-presenting cell-dependent stimulation (45). There are two essential signals involved in T cell activation. The first signal is antigen-specific interaction between MHC on the APC surface and TCRs on the T cell surface. The second signal is nonspecific interaction between CD80/CD86 on the APC surface and CD28 on the T cell. Our scRNA data denotes that hemodialysis elicits substantially diverse effects on different types of major histocompatibility complexes, as reflected by decreased expression of MHCII presentation genes (e.g., *HLA-DRB1*, *HLA-DQA2*, *HLA-DQA1*, *HLA-DPB1)* and increased expression of MHCI presentation genes (*B2M*, *CD74*, *HSPA6*, *HSPA1B*, *HSPA1A*). Hence, we propose that the dysfunction of the TCR downstream pathways in CD4+ T cells may be partly attributed to the impaired TCR-pMHCII interaction. Although some studies have suggested that a disturbance in CD80/CD86-CD28 could be responsible for the impaired immune function in HD patients (46), we found no evidence to support this finding. Monocytes are preactivated and induce hypersecretion of IL-6, IL-12, and TNFα during the HD procedure (47), leading to its spontaneous apoptosis, senescence, and non-responsiveness to HBV vaccination. On the other hand, IL-10, which is produced by monocytes, decreases monocyte MHCII expression and promotes differentiation of regulatory T cells (48). In addition to TCR-pMHCII, the enhanced HVEM-BTLA signaling and diminished TGFB-TGFBR and IL16-CD4 signalings between APCs and CD4+ T cells may represent other factors involved in the regulation of TCR downstream pathways in HD patients. Although the effects of these signalings on dialysis-derived T cells are rarely reported or observed, BTLA and TGFBR signalings have been well characterized and have been shown to be important in T and B cell function (49). Hemodialysis has relatively little impact on immune pathways of other PBMC subpopulations (B cell and NK), at least from a transcriptomic perspective, compared to the complex pathway abnormalities in CD4+ T cells. It should be recognized that the hemodialysis procedure also poses a threat to other PBMC subsets, as a diffuse reduction of B-cell subpopulations and increased activity of cytotoxic effector NK cells have been revealed in the long-term HD (10, 50). We can review the transcriptomic profile of each dialysis-derived PBMC subset in **Supplementary Table 4**.

Our study is limited by the lack of validation from functional experiments. Therefore, the present study has not yet confirmed the actual effects of the disrupted communication signalings on T cell function in HD patients. In addition, patients with relatively early dialysis years or patients with ESRD not on hemodialysis are not recruited for this study, making it unclear whether the differential results are attributable to ESRD or hemodialysis itself.

## Conclusion

By combining scRNA-seq and scATAC-seq, we constructed gene expression profiles and intercellular communication patterns of PBMC subpopulations in chronic hemodialysis patients. We discovered that hemodialysis significantly caused abnormalities in the PI3K-Akt-mTOR, MAPK, TNF, NF-κB pathways in CD4+ T cell subsets, which might be partly ascribed to defective TCR-pMHCII, TGFBR, BTLA, and CD4 signalings. We highlighted the decreased levels of AP-1 family TFs (*JUN*, *JUND*, *FOS*, and *FOSB*) in dialysis-derived CD4+ T cells, which were associated with decreased chromosomal accessibility of the *JUN*, *JUND*, *FOS*, and *FOSB* regions.

## Data Availability Statement

The datasets presented in this study can be found in online repositories. The names of the repository/repositories and accession number(s) can be found below: https://www.ncbi.nlm.nih.gov/geo/query/acc.cgi?acc=GSE158280; GSA (https://ngdc.cncb.ac.cn/gsa-human/) with number ID: HRA001999.

## Ethics Statement

The present study was conducted following the principles of the Declaration of Helsinki and was authorized by the Ethics Board of Shenzhen People’s Hospital. All participants have signed informed consent forms. The patients/participants provided their written informed consent to participate in this study.

## Author Contributions

YD, DT, and FL designed the experiments. HW, JD, and HY performed the experiments and wrote the manuscript. NH, LY, and WD performed the computational analysis. XZ and KW collected and assembled data. All authors critically revised the manuscript. All authors contributed to the article and approved the submitted version.

## Funding

This work was supported by The Science and Technology Plan of Shenzhen (NO.JCYJ20190807153405508), Shenzhen Fund for Guangdong Provincial High-level Clinical Key Specialties (NO.SZGSP001), Key Renal Laboratory of Shenzhen, Department of Nephrology (ZDSYS201504301616234), The key-area Research in General Colleges and Universities of Guangdong Province (2021ZDZX2042).

## Conflict of Interest

The authors declare that the research was conducted in the absence of any commercial or financial relationships that could be construed as a potential conflict of interest.

## Publisher’s Note

All claims expressed in this article are solely those of the authors and do not necessarily represent those of their affiliated organizations, or those of the publisher, the editors and the reviewers. Any product that may be evaluated in this article, or claim that may be made by its manufacturer, is not guaranteed or endorsed by the publisher.
