# Supplementary material for: Single-Cell RNA and ATAC Sequencing Reveal Hemodialysis-Related Immune Dysregulation of Circulating Immune Cell Subpopulations
Source: Front Immunol. 2022 May 26;13:878226. doi: 10.3389/fimmu.2022.878226 (PMC9205630; doi:10.3389/fimmu.2022.878226)
Supplement: Supplementary file 1 [file DataSheet_1.docx]

**Supplementary Figures**


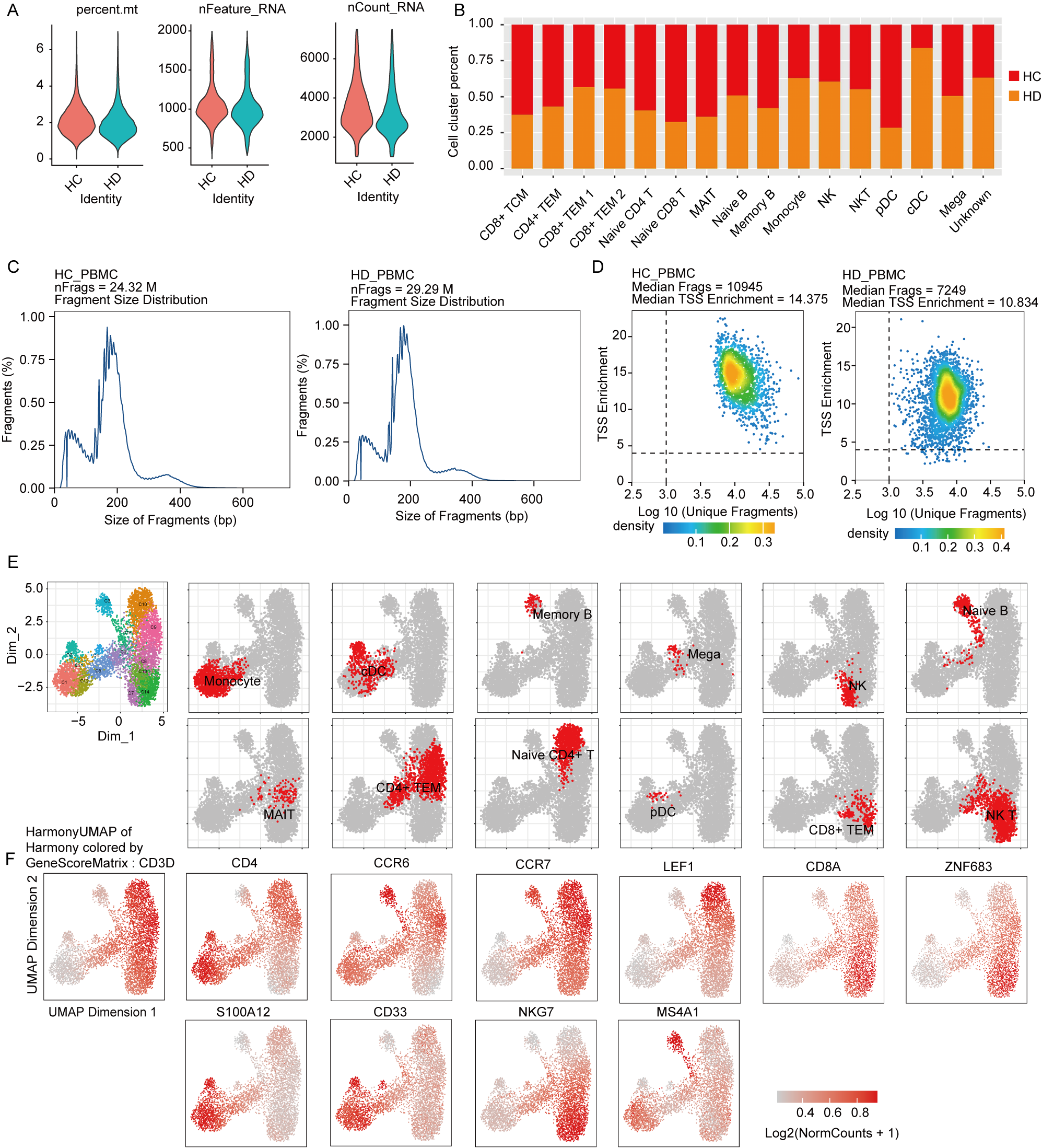


Supplementary Figure 1. Quality control and cell-type annotation of scRNA-seq and scATAC-seq data. (A) Violin plots showing the number of genes, unique molecular identifiers, and the percentage of mitochondrial genes in HC and HD groups. (B) Cell abundance in each cell type across the HC-PBMC and HD-PBMC libraries. (C) Histogram of the distribution of fragment lengths in reads from the HC_PBMC and HD_PBMC groups. (D) TSS enrichment scores for HC_PBMC and HD_PBMC groups. (E) Alignment of scATAC-seq cells with scRNA-seq cells by comparing the gene expression matrix of scRNA-seq data with the gene score matrix of scATAC-seq data. (F) UMAP plots of cell markers for identifying scATAC-seq cell subpopulations. Abbreviation: PBMC, peripheral blood mononuclear cell; HD, hemodialysis; HC, healthy control; UMAP, uniform manifold approximation and projection for dimension reduction; TSS, transcription start site.


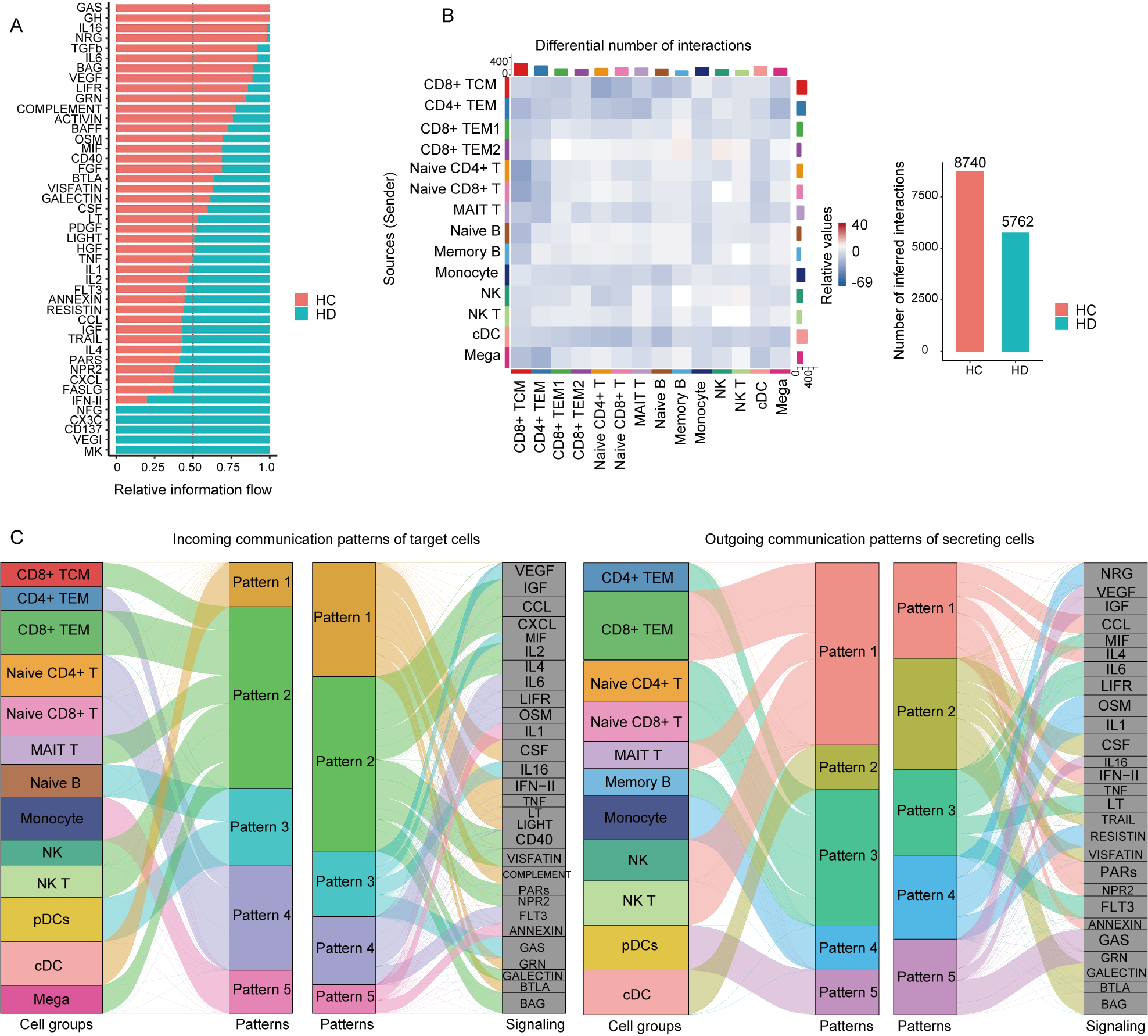


Supplementary Figure 2. CellChat analysis of the communication between PBMC subpopulations. (A) All significant signaling pathways were ranked based on their differences of overall information flow within the inferred networks between HC_PBMC and HD_PBMC. (B) Heatmap showing the differential number of interactions between HC and HD groups. (C) Alluvial plots show the incoming signaling patterns of target cells (left) and the outgoing signaling patterns of secreting cells (right) in healthy subjects. The thickness of the flow represents the contribution of the cell group or signaling pathway to each corresponding pattern. Abbreviation: PBMC, peripheral blood mononuclear cell; HD, hemodialysis; HC, healthy control.


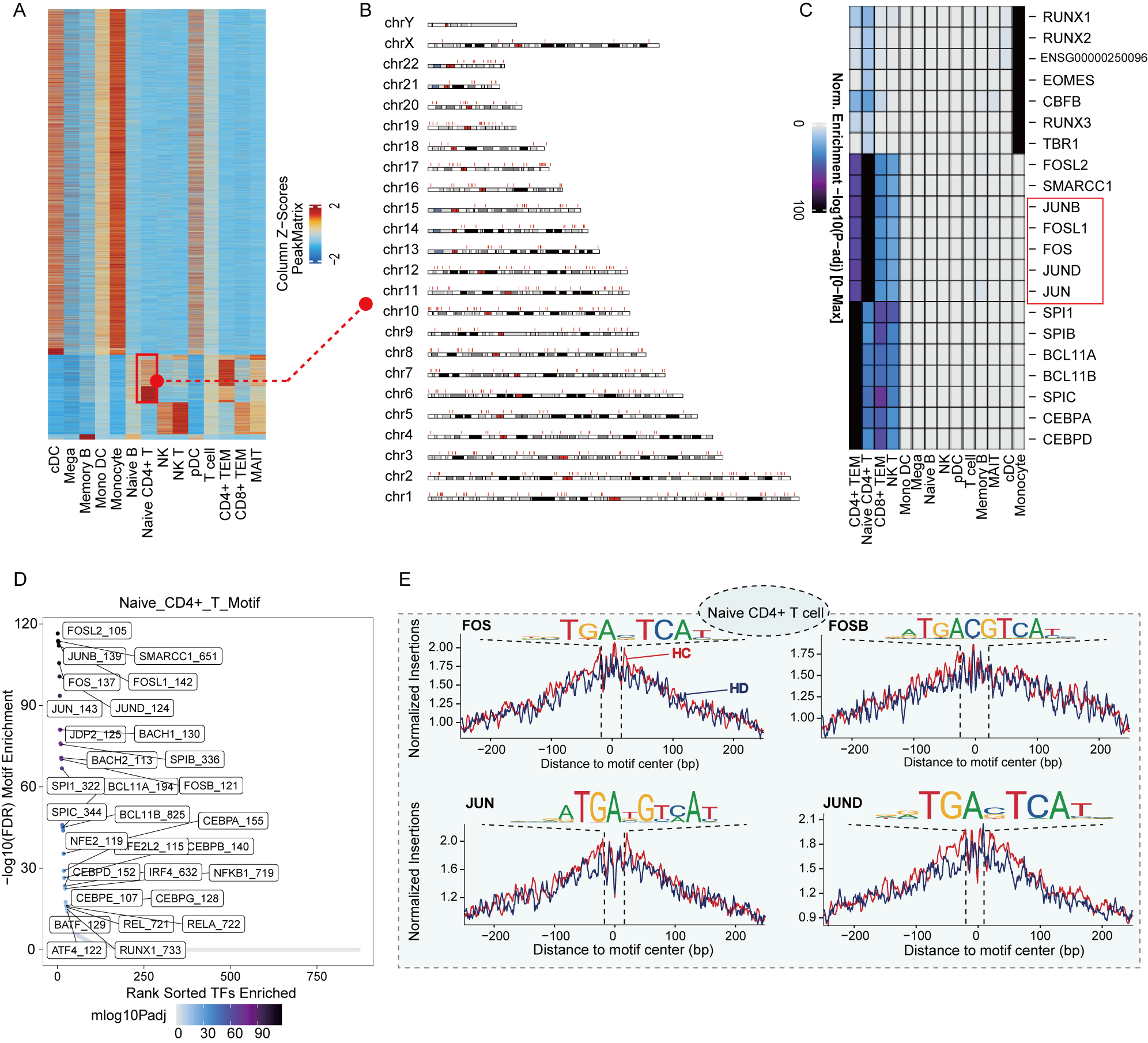
Supplementary Figure 3. Single-cell ATAC analysis of naive CD4+ T cells. (A) Heatmap showing the Cell-type-specific peaks. (B) The chromosome location of differentially expressed peaks in naive CD4+ T cells. (C) Cell-type-specific TF motifs identification. (D) Motif enrichment of differentially expressed peaks in naive CD4+ T cells. (E) Footprint analysis of FOS, FOSB, JUN, and JUND in naive CD4+ T cells.


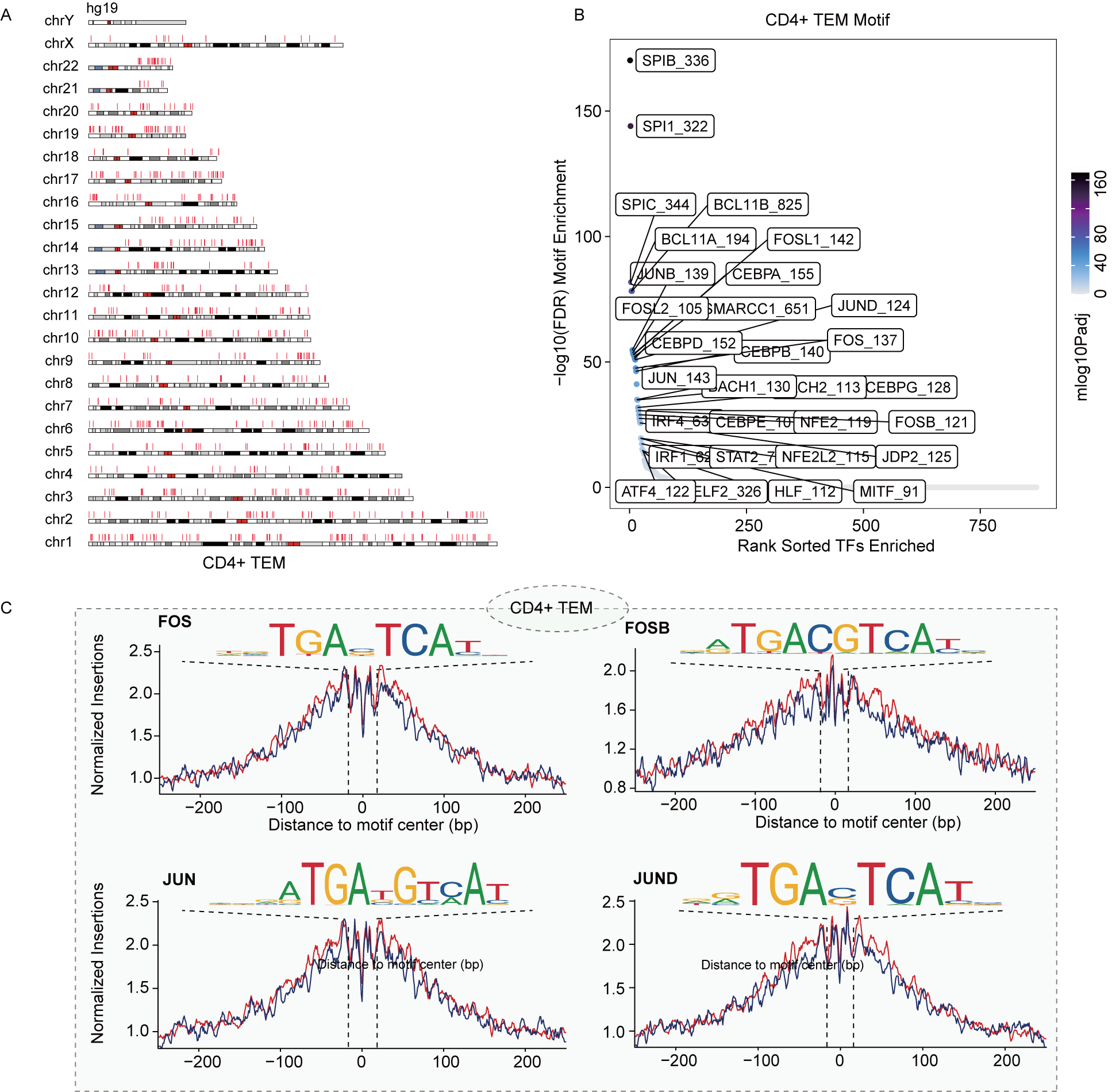
Supplementary Figure 4. Single-cell ATAC analysis of CD4+ TEM cells. (A) The chromosome location of differentially expressed peaks in CD4+ TEM cells. (B) Motif enrichment of differentially expressed peaks in CD4+ TEM cells. (C) Footprint analysis of FOS, FOSB, JUN, and JUND in CD4+ TEM cells.
